# Supplementary material for: Treatment sequences of patients with advanced colorectal cancer and use of second-line FOLFIRI with antiangiogenic drugs in Japan: A retrospective observational study using an administrative database
Source: PLoS One. 2021 Feb 8;16(2):e0246160. doi: 10.1371/journal.pone.0246160 (PMC7870079; doi:10.1371/journal.pone.0246160)

S1a Fig. Treatment sequences for the first-line population. *RAS*, rat sarcoma viral oncogene homolog; CRC, colorectal cancer; EGFRab, anti-epidermal growth factor receptor antibody (includes panitumumab, cetuximab); FP, fluoropyrimidine (includes capecitabine, 5-fluorouracil, combination tegafur/gimestat/potassium otastat [S-1], combination uracil/tegafur [UFT]); OXALI, oxaliplatin; anti-VEGF, anti-vascular endothelial growth factor receptor (includes the antiangiogenic drugs bevacizumab, ramucirumab, aflibercept beta); IRI, irinotecan; FTD/TPI, trifluridine/tipiracil; REG, regorafenib.

Presumed *RAS*-mutant CRC (no anti-EGFR antibody prescription during the analysis period)

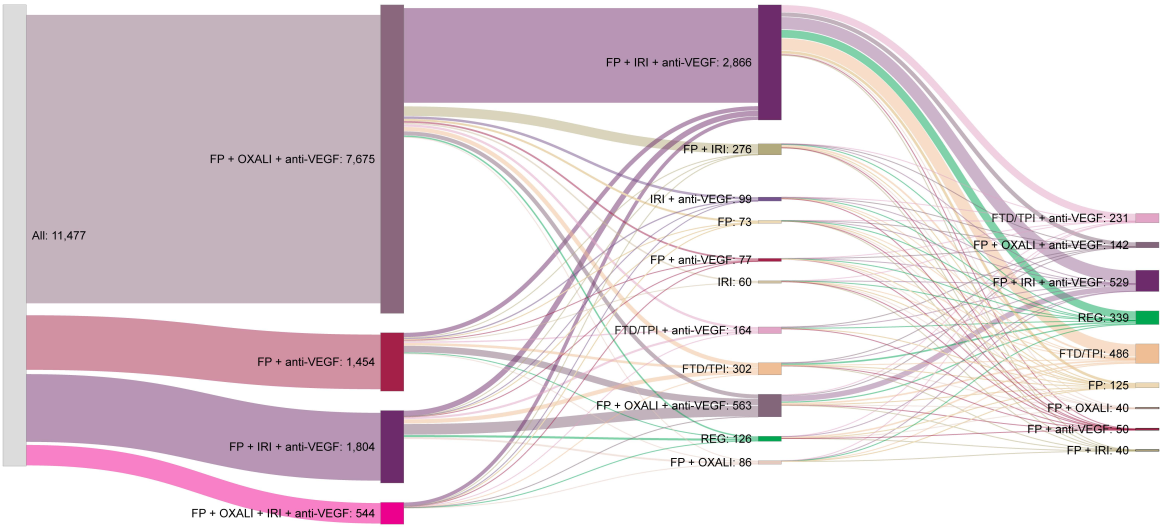

Presumed *RAS*-wild type CRC (anti-EGFR antibody prescription during the analysis period)

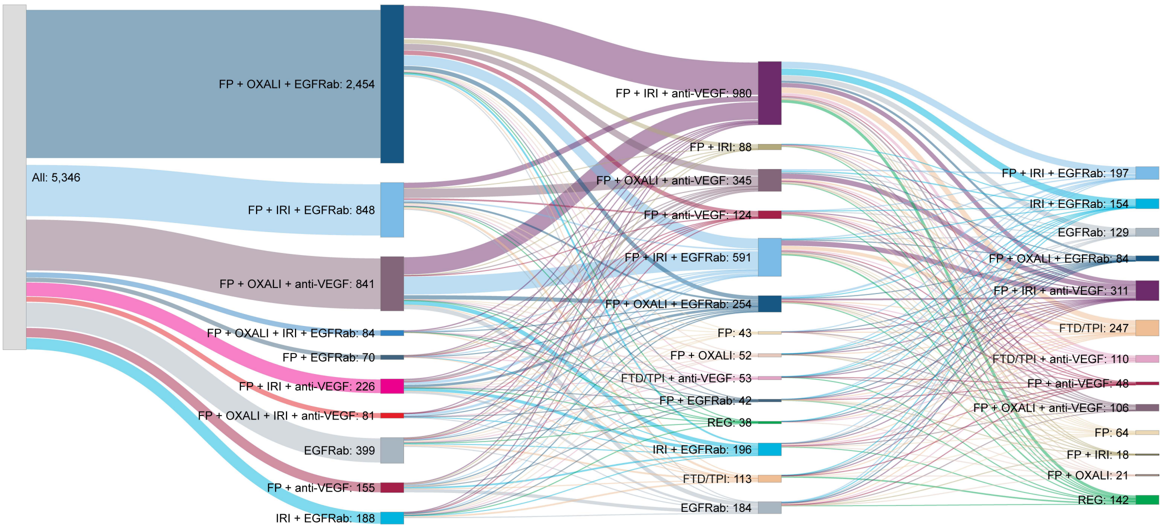

Supplement: S1A Fig — (PDF) [file pone.0246160.s001.pdf]
