# Supplementary material for: Treatment sequences of patients with advanced colorectal cancer and use of second-line FOLFIRI with antiangiogenic drugs in Japan: A retrospective observational study using an administrative database
Source: PLoS One. 2021 Feb 8;16(2):e0246160. doi: 10.1371/journal.pone.0246160 (PMC7870079; doi:10.1371/journal.pone.0246160)
Supplement: S1A Table — (PDF) [file pone.0246160.s003.pdf]

**S1a Table. Five most common treatment regimens in the first, second, and third lines for patients who started systemic therapy including biologics without prior evidence of early recurrence (first-line population).**

| Line of therapy | No anti-EGFR antibody prescription during the AP (presumed *RAS*-mutant CRC) | | | Anti-EGFR antibody prescription during the AP (presumed *RAS*-wild type CRC) | |
|---|---|---|---|---|---|
| | Treatment | n (%) | | Treatment | n (%) |
| 1st line | | | 1st line | | |
| (n=11,756) | BEV, FOLFOX | 3,605 (30.7) | (n=5,389) | PANI, FOLFOX | 1,878 (34.9) |
| | BEV, CAPOX | 2,950 (25.1) | | PANI, FOLFIRI | 498 (9.2) |
| | BEV, FOLFIRI | 1,164 (9.9) | | BEV, FOLFOX | 399 (7.4) |
| | BEV, SOX | 1,074 (9.1) | | CET, FOLFOX | 362 (6.7) |
| | BEV, CAPE | 871 (7.4) | | BEV, CAPOX | 339 (6.3) |
| 2nd line | | | 2nd line | | |
| (n=4,897) | BEV, FOLFIRI | 972 (19.9) | (n=3,239) | BEV, FOLFIRI | 488 (15.1) |
| | RAM, FOLFIRI | 832 (17.0) | | PANI, FOLFIRI | 402 (12.4) |
| | BEV, IRIS | 667 (13.6) | | BEV, FOLFOX | 230 (7.1) |
| | FTD/TPI | 301 (6.2) | | RAM, FOLFIRI | 229 (7.1) |
| | AFL FOLFIRI | 271 (5.5) | | PANI, FOLFOX | 184 (5.7) |
| 3rd line | | | 3rd line | | |
| (n=2,060) | FTD/TPI | 484 (23.5%) | (n=1,734) | FTD/TPI | 247 (14.2) |
| | REG | 343 (16.7%) | | REG | 148 (8.5) |
| | BEV, FTD/TPI | 233 (11.3%) | | PANI, FOLFIRI | 138 (8.0) |
| | RAM FOLFIRI | 212 (10.3%) | | RAM, FOLFIRI | 132 (7.6) |
| | AFL, FOLFIRI | 108 (5.2%) | | PANI, IRI | 112 (6.5) |

EGFR, epidermal growth factor receptor; AP, analysis period; *RAS*, rat sarcoma viral oncogene homolog; CRC, colorectal cancer; BEV, bevacizumab; FOLFOX, leucovorin, 5-fluorouracil, and oxaliplatin; CAPOX, capecitabine and oxaliplatin; FOLFIRI, leucovorin, 5-fluorouracil, and irinotecan; SOX, S-1 and oxaliplatin; CAPE, capecitabine; PANI, panitumumab; CET, cetuximab; RAM, ramucirumab; IRIS, S-1 and irinotecan; FTD/TPI, trifluridine/tipiracil; AFL, aflibercept beta; REG, regorafenib; IRI, irinotecan.
