# Supplementary material for: Treatment sequences of patients with advanced colorectal cancer and use of second-line FOLFIRI with antiangiogenic drugs in Japan: A retrospective observational study using an administrative database
Source: PLoS One. 2021 Feb 8;16(2):e0246160. doi: 10.1371/journal.pone.0246160 (PMC7870079; doi:10.1371/journal.pone.0246160)
Supplement: S1B Table — (PDF) [file pone.0246160.s004.pdf]

S1b Table. Five most common treatment regimens in the adjuvant, second, and third lines for patients who underwent colorectal surgery and experienced early recurrence after adjuvant therapy (early recurrence population).

Line of therapy	No anti-EGFR antibody prescription during the AP (presumed *RAS*-mutant CRC)			Anti-EGFR antibody prescription during the AP (presumed *RAS*-wild type CRC)	
	Treatment	n (%)		Treatment	n (%)
Adjuvant			Adjuvant		
(n=1,362)	CAPOX	378 (27.8)	(n=330)	CAPOX	128 (38.8)
	CAPE	294 (21.6)		CAPE	64 (19.4)
	UFT	272 (20.0)		UFT	53 (16.1)
	FOLFOX	183 (13.4)		FOLFOX	42 (12.7)
	S-1	140 (10.3)		S-1	25 (7.6)
2nd line			2nd line		
(n=1,362)	BEV, CAPOX	203 (14.9)	(n=330)	PANI, FOLFIRI	53 (16.1)
	BEV, FOLFOX	197 (14.5)		PANI, FOLFOX	46 (13.9)
	BEV, FOLFIRI	190 (14.0)		BEV, CAPOX	23 (7.0)
	CAPOX	160 (11.8)		BEV, FOLFIRI	21 (6.4)
	BEV, IRIS	109 (8.0)		BEV, FOLFOX	19 (5.8)
3rd line			3rd line		
(n=576)	RAM, FOLFIRI	87 (15.1)	(n=235)	PANI, FOLFIRI	29 (12.3)
	BEV, IRIS	73 (12.7)		PANI, FOLFOX	18 (7.7)
	BEV, FOLFIRI	72 (12.5)		PANI, IRI	17 (7.2)
	FTD/TPI	47 (8.2)		BEV, FOLFIRI	17 (7.2)
	REG	33 (5.7)		FTD/TPI	14 (6.0)

EGFR, epidermal growth factor receptor; AP, analysis period; *RAS*, rat sarcoma viral oncogene homolog; CRC, colorectal cancer; CAPOX, capecitabine and oxaliplatin; CAPE, capecitabine; UFT, uracil/tegafur; FOLFOX, leucovorin, 5-fluorouracil, and oxaliplatin; S-1, tegafur/gimestat/potassium otastat; BEV, bevacizumab; FOLFIRI, leucovorin, 5-fluorouracil, and irinotecan; IRIS, S-1 and irinotecan; PANI, panitumumab; RAM, ramucirumab; FTD/TPI, trifluridine/tipiracil; REG, regorafenib; IRI, irinotecan.
