# Supplementary material for: Treatment sequences of patients with advanced colorectal cancer and use of second-line FOLFIRI with antiangiogenic drugs in Japan: A retrospective observational study using an administrative database
Source: PLoS One. 2021 Feb 8;16(2):e0246160. doi: 10.1371/journal.pone.0246160 (PMC7870079; doi:10.1371/journal.pone.0246160)
Supplement: S2 Table — (PDF) [file pone.0246160.s005.pdf]

S2 Table. Baseline demographics and clinical characteristics of patients with CRC in the combined first-line and early recurrence populations who received FOLFIRI in combination with antiangiogenic drugs.

Variable	Second-line therapy			
	FOLFIRI + any angiogenic drug N=3,138	FOLFIRI + bevacizumab N=1,671	FOLFIRI + ramucirumab N=1,095	FOLFIRI + aflibercept beta N=372
Hospital size by number of beds, n (%)				
<200 beds	202 (6.4)	136 (8.1)	44 (4.0)	22 (5.9)
200–499 beds	1,826 (58.2)	983 (58.8)	662 (60.5)	181 (48.7)
≥500 beds	1,110 (35.4)	552 (33.0)	389 (35.5)	169 (45.4)
Designated cancer hospital, n (%)	2,253 (71.8)	1,169 (70.0)	788 (72.0)	296 (79.6)
Medical department where first 2nd-line therapy was prescribed, n (%)				
Internal medicine	878 (28.0)	523 (31.3)	247 (22.6)	108 (29.0)
Surgery	2,223 (70.8)	1,136 (68.0)	833 (76.1)	254 (68.3)
Others	34 (1.1)	11 (0.7)	13 (1.2)	10 (2.7)
Unknown	3 (0.1)	1 (0.1)	2 (0.2)	0
Age at start of 2nd line, mean (SD)	66.0 (10.3)	66.3 (10.1)	65.6 (10.7)	65.5 (10.0)
Age ≥70 years at start of 2nd line, n (%)	1,272 (40.5)	688 (41.2%)	446 (40.7)	138 (37.1)
Sex: male, n (%)	1,821 (58.0)	987 (59.1)	609 (55.6)	225 (60.5)

Variable	Second-line therapy			
	**FOLFIRI + any angiogenic drug N=3,138**	**FOLFIRI + bevacizumab N=1,671**	**FOLFIRI + ramucirumab N=1,095**	**FOLFIRI + aflibercept beta N=372**
Presumed *RAS*-wild type CRC (received anti-EGFR antibody at any time), n (%)	830 (26.5)	509 (30.5)	232 (21.2)	89 (23.9)
Left-sided colorectal cancer, n (%)	2,162 (68.9)	1,175 (70.3)	726 (68.3)	261 (70.2)
Right-sided colorectal cancer, n (%)	1,077 (34.3)	556 (33.3)	398 (36.4)	123 (33.1)
BMI, kg/m^2, mean (SD) [a]	22.3 (3.7)	22.2 (3.8)	22.4 (3.6)	22.0 (3.5)
ADL – independent, n (%) [b]	2,534 (92.1)	1,352 (91.0)	887 (93.7)	295 (92.8)
ADL – not independent. n (%) [b]	217 (7.9)	134 (9.0)	60 (6.3)	23 (7.2)
Included in 1st-line or adjuvant therapy, n (%)				
Bevacizumab	2,306 (73.5)	1,099 (65.8)	905 (82.7)	302 (81.2)
Anti-EGFR antibody	585 (18.6)	365 (21.8)	160 (14.6)	60 (16.1)
Fluoropyrimidine, oral	781 (24.9)	358 (21.4)	307 (28.0)	116 (31.2)
Fluoropyrimidine, i.v.	2,465 (78.6)	1,359 (81.3)	831 (75.9)	275 (73.9)
Irinotecan	342 (10.9)	80 (4.8)	173 (15.8)	89 (23.9)
FOLFOXIRI	113 (3.6)	9 (0.5)	58 (5.3)	46 (12.4)
Treatment duration of 1st-line or adjuvant, days, median (IQR)	176 (99–284)	164 (92–260)	187 (106–293)	225 (120–343)

CRC, colorectal cancer; FOLFIRI, leucovorin, fluorouracil, and irinotecan; SD, standard deviation; *RAS*, rat sarcoma viral oncogene homolog; EGFR, endothelial growth factor receptor; BMI, body mass index; ADL, activities of daily living; i.v., intravenous; FOLFOXIRI, leucovorin, fluorouracil, oxaliplatin, and irinotecan; IQR, interquartile range.

[a] Any antiangiogenic drug, N=2,774; bevacizumab, N=1,500; ramucirumab, N=952, aflibercept beta, N=322.

[b] Any antiangiogenic drug, N=2,751; bevacizumab, N=1,486; ramucirumab, N=947, aflibercept beta, N=318.
