# Supplementary material for: Treatment sequences of patients with advanced colorectal cancer and use of second-line FOLFIRI with antiangiogenic drugs in Japan: A retrospective observational study using an administrative database
Source: PLoS One. 2021 Feb 8;16(2):e0246160. doi: 10.1371/journal.pone.0246160 (PMC7870079; doi:10.1371/journal.pone.0246160)
Supplement: S3A Table — (DOCX) [file pone.0246160.s006.docx]

**S3a Table. Prescription characteristics and treatment continuation in the FOLFIRI plus bevacizumab population (second-line treatment).**

| **Variable** | **Value** |
| --- | --- |
| Duration of 2^nd^-line treatment with bevacizumab (months) ^a^ | N=1,671 |
| Mean (SE) | 7.5 (0.22) |
| Median (95% CI) | 5.1 (4.8–5.5) |
| Prescription characteristics and transition rate in 2^nd^-line treatment with FOLFIRI plus bevacizumab | N=1,293 ^b^ |
| Patients who transitioned to 3^rd^-line treatment, n (%) | 893 (69.1) |
| Number of bevacizumab prescriptions, median (IQR) | 6 (3–11) |
| Patients with bevacizumab dose reductions, n (%) | 95 (7.4) |
| Patients with bevacizumab prescription gaps ≥21 days, n (%) | 705 (54.5) |
| Patients who used bevacizumab once, n (%) | 111 (8.6) |

FOLFIRI, leucovorin, fluorouracil, and irinotecan; SE, standard error; CI, confidence interval; IQR, interquartile range.

^a^ Duration was estimated using the Kaplan-Meier method. The mean survival time and its standard error were underestimated because the largest observation was censored and the estimation was restricted to the largest event time.

^b^ Patients with data available ≥60 days after the end of second-line therapy or patients who transitioned to third-line therapy were included in this analysis.
