# Supplementary material for: Treatment sequences of patients with advanced colorectal cancer and use of second-line FOLFIRI with antiangiogenic drugs in Japan: A retrospective observational study using an administrative database
Source: PLoS One. 2021 Feb 8;16(2):e0246160. doi: 10.1371/journal.pone.0246160 (PMC7870079; doi:10.1371/journal.pone.0246160)
Supplement: S3B Table — (DOCX) [file pone.0246160.s007.docx]

**S3b Table. Prescription characteristics and treatment continuation in the FOLFIRI plus ramucirumab population (second-line treatment).**

| **Variable** | **Value** |
| --- | --- |
| Duration of 2^nd^-line treatment with ramucirumab (months) ^a^ | N=1,095 |
| Mean (SE) | 5.7 (0.20) |
| Median (95% CI) | 3.8 (3.5–4.0) |
| Prescription characteristics and transition rate in 2^nd^-line treatment with FOLFIRI plus ramucirumab | N=856 ^b^ |
| Patients who transitioned to 3^rd^-line treatment, n (%) | 544 (63.6) |
| Number of ramucirumab prescriptions, median (IQR) | 4 (2–7.3) |
| Patients with ramucirumab dose reductions, n (%) | 151 (17.6) |
| Patients with ramucirumab prescription gaps ≥21 days, n (%) | 454 (53.0) |
| Patients who used ramucirumab once, n (%) | 135 (15.8) |

FOLFIRI, leucovorin, fluorouracil, and irinotecan; SE, standard error; CI, confidence interval; IQR, interquartile range.

^a^ Duration was estimated using the Kaplan-Meier method. The mean survival time and its standard error were underestimated because the largest observation was censored and the estimation was restricted to the largest event time.

^b^ Patients with data available ≥60 days after the end of second-line therapy or patients who transitioned to third-line therapy were included in this analysis.
