# Supplementary material for: Treatment sequences of patients with advanced colorectal cancer and use of second-line FOLFIRI with antiangiogenic drugs in Japan: A retrospective observational study using an administrative database
Source: PLoS One. 2021 Feb 8;16(2):e0246160. doi: 10.1371/journal.pone.0246160 (PMC7870079; doi:10.1371/journal.pone.0246160)
Supplement: S3C Table — (DOCX) [file pone.0246160.s008.docx]

**S3c Table. Prescription characteristics and treatment continuation in the FOLFIRI plus aflibercept beta population (second-line treatment).**

| **Variable** | **Value** |
| --- | --- |
| Duration of 2^nd^-line treatment with aflibercept beta (months) ^a^ | N=372 |
| Mean (SE) | 5.6 (0.31) |
| Median (95% CI) | 4.0 (3.5–4.9) |
| Prescription characteristics and transition rate in 2^nd^-line treatment with FOLFIRI plus aflibercept beta | N=281 ^b^ |
| Patients who transitioned to 3^rd^-line treatment, n (%) | 171 (60.9) |
| Number of aflibercept beta prescriptions, median (IQR) | 4 (2–9) |
| Patients with aflibercept beta dose reductions, n (%) | 49 (17.4) |
| Patients with aflibercept beta prescription gaps ≥21 days, n (%) | 151 (53.7) |
| Patients who used aflibercept beta once, n (%) | 37 (13.2) |

FOLFIRI, leucovorin, fluorouracil, and irinotecan; SE, standard deviation; CI, confidence interval; IQR, interquartile range.

^a^ Duration was estimated using the Kaplan-Meier method.

^b^ Patients with data available ≥60 days after the end of second-line therapy or patients who transitioned to third-line therapy were included in this analysis.
