# Supplementary material for: Treatment sequences of patients with advanced colorectal cancer and use of second-line FOLFIRI with antiangiogenic drugs in Japan: A retrospective observational study using an administrative database
Source: PLoS One. 2021 Feb 8;16(2):e0246160. doi: 10.1371/journal.pone.0246160 (PMC7870079; doi:10.1371/journal.pone.0246160)
Supplement: S4 Table — (PDF) [file pone.0246160.s009.pdf]

S4 Table. Concomitant procedures and medications received on the first date of a second-line antiangiogenic therapy in the FOLFIRI plus antiangiogenic drug subpopulation.

Concomitant procedures and medications received, n (%)	FOLFIRI + any angiogenic drug (N=3,138)	FOLFIRI + bevacizumab (N=1,671)	FOLFIRI + ramucirumab (N=1,095)	FOLFIRI + aflibercept beta (N=372)
Qualitative proteinuria tests	1,169 (37.3%)	561 (33.6%)	452 (41.3%)	156 (41.9%)
Quantitative proteinuria tests	222 (7.1%)	62 (3.7%)	100 (9.1%)	60 (16.1%)
No proteinuria tests	1,960 (62.5%)	1,107 (66.3%)	639 (58.4%)	214 (57.5%)
Antihypertensives	664 (21.2%)	329 (19.7%)	242 (22.1%)	93 (25.0%)
Anticoagulants	67 (2.1%)	27 (1.6%)	30 (2.7%)	10 (2.7%)
Anticholinergics	337 (10.7%)	155 (9.3%)	121 (11.1%)	61 (16.4%)

FOLFIRI, leucovorin, fluorouracil, and irinotecan.
