# Supplementary material for: Treatment sequences of patients with advanced colorectal cancer and use of second-line FOLFIRI with antiangiogenic drugs in Japan: A retrospective observational study using an administrative database
Source: PLoS One. 2021 Feb 8;16(2):e0246160. doi: 10.1371/journal.pone.0246160 (PMC7870079; doi:10.1371/journal.pone.0246160)
Supplement: S5A Table — (PDF) [file pone.0246160.s010.pdf]

S5a Table. Multivariate Cox regression analysis for the factors associated with overall treatment continuation from the start of second-line therapy to the end of all antitumor drug therapies in the FOLFIRI plus antiangiogenic drug subpopulation, for patients with left-sided CRC.

Covariate	Hazard ratio	95% CI	p-value
Designated cancer hospital (yes vs no)	1.15	1–1.32	0.0458
≥70 vs <70 years at start of 2nd-line therapy	1.03	0.9–1.17	0.6856
Sex: male vs female	1	0.88–1.14	0.9654
Presumed *RAS*-wild type (yes vs no)	0.59	0.51–0.68	<0.0001
BMI ≤18.5 kg/m^2 vs >18.5 kg/m^2	1.37	1.16–1.63	0.0003
ADL (not independent vs independent)	1.35	1.09–1.66	0.0053
Oral fluoropyrimidine in previous line of therapy (yes vs no)	0.77	0.67–0.9	0.0009
Irinotecan in previous line (yes vs no)	1.12	0.91–1.39	0.2798
Duration of previous line of therapy ≥180 days vs <180 days	0.87	0.77–0.99	0.0356
Early recurrence (yes vs no)	0.6	0.47–0.77	<0.0001
Concomitant procedures and medications during 2nd-line therapy (yes vs no)			
Qualitative proteinuria tests	0.65	0.57–0.75	<0.0001
Quantitative proteinuria tests	0.94	0.8–1.12	0.503
Antihypertensives	0.85	0.75–0.96	0.0085
Anticholinergics	0.89	0.77–1.03	0.123
Anticoagulants	0.93	0.7–1.22	0.576

FOLFIRI, leucovorin, fluorouracil, and irinotecan; CRC, colorectal cancer; CI, confidence interval; *RAS*, rat sarcoma viral oncogene homolog; BMI, body mass index; ADL, activities of daily living; EGFR, endothelial growth factor receptor.

1,885 patients who started FOLFIRI plus antiangiogenic drug as second-line and had ADL and BMI data available from baseline period before second-line and with left-sided CRC diagnosis were included in this analysis.
