# Supplementary material for: Treatment sequences of patients with advanced colorectal cancer and use of second-line FOLFIRI with antiangiogenic drugs in Japan: A retrospective observational study using an administrative database
Source: PLoS One. 2021 Feb 8;16(2):e0246160. doi: 10.1371/journal.pone.0246160 (PMC7870079; doi:10.1371/journal.pone.0246160)
Supplement: S5B Table. Multivariate Cox regression analysis for the factors associated with overall treatment continuation from the start of second-line therapy to the end of all antitumor drug therapies in the FOLFIRI plus antiangiogenic drug subpopulation, for patients with right-sided CRC — (PDF) [file pone.0246160.s011.pdf]

S5b Table. Multivariate Cox regression analysis for the factors associated with overall treatment continuation from the start of second-line therapy to the end of all antitumor drug therapies in the FOLFIRI plus antiangiogenic drug subpopulation, for patients with right-sided CRC.

Covariate	Hazard ratio	95% CI	p-value
Designated cancer hospital (yes vs no)	1.1	0.91–1.32	0.3296
≥70 vs <70 years at start of 2nd-line therapy	1.08	0.91–1.28	0.3562
Sex: male vs female	1.11	0.94–1.31	0.2279
Presumed *RAS*-wild type (yes vs no)	0.71	0.58–0.88	0.0016
BMI ≤18.5 kg/m^2 vs >18.5 kg/m^2	1.18	0.94–1.47	0.1571
ADL (not independent vs independent)	1.3	0.97–1.73	0.0756
Oral fluoropyrimidine in previous line of therapy (yes vs no)	0.92	0.76–1.12	0.3961
Irinotecan in previous line (yes vs no)	1.25	0.93–1.68	0.1361
Duration of previous line of therapy ≥180 days vs <180 days	1.01	0.85–1.2	0.9282
Early recurrence (yes vs no)	0.79	0.6–1.06	0.1141
Concomitant procedures and medications during 2nd-line therapy (yes vs no)			
Qualitative proteinuria tests	0.85	0.7–1.03	0.1055
Quantitative proteinuria tests	0.81	0.65–1.02	0.0684
Antihypertensives	0.98	0.83–1.17	0.8598
Anticholinergics	0.95	0.77–1.18	0.6727
Anticoagulants	0.95	0.65–1.39	0.7892

FOLFIRI, leucovorin, fluorouracil, and irinotecan; CRC, colorectal cancer; CI, confidence interval; *RAS*, rat sarcoma viral oncogene homolog; BMI, body mass index; ADL, activities of daily living; EGFR, endothelial growth factor receptor.

951 patients who started FOLFIRI plus antiangiogenic drug as second-line and had ADL and BMI data available from baseline period before second-line and with right-sided CRC diagnosis were included in this analysis.
