# Supplementary material for: Treatment sequences of patients with advanced colorectal cancer and use of second-line FOLFIRI with antiangiogenic drugs in Japan: A retrospective observational study using an administrative database
Source: PLoS One. 2021 Feb 8;16(2):e0246160. doi: 10.1371/journal.pone.0246160 (PMC7870079; doi:10.1371/journal.pone.0246160)
Supplement: S5C Table — (PDF) [file pone.0246160.s012.pdf]

S5c Table. Multivariate Cox regression analysis for the factors associated with overall treatment continuation from the start of second-line therapy to the end of all antitumor drug therapies in the FOLFIRI plus antiangiogenic drug subpopulation, for patients with presumed *RAS*-wild type CRC.

Covariate	Hazard ratio	95% CI	p-value
Designated cancer hospital (yes vs no)	0.84	0.67–1.06	0.1404
≥70 vs <70 years at start of 2nd-line therapy	0.86	0.68–1.07	0.1736
Sex: male vs female	1.01	0.81–1.26	0.9285
Left-sided CRC (yes vs no)	0.67	0.53–0.84	0.0007
BMI ≤18.5 kg/m^2 vs >18.5 kg/m^2	1.29	0.97–1.73	0.08
ADL (not independent vs independent)	1.9	1.33–2.72	0.0004
Oral fluoropyrimidine in previous line of therapy (yes vs no)	0.74	0.55–1.01	0.0559
Irinotecan in previous line (yes vs no)	0.9	0.66–1.23	0.5097
Duration of previous line of therapy ≥180 days vs <180 days	0.89	0.72–1.1	0.2881
Early recurrence (yes vs no)	0.6	0.32–1.13	0.1133
Concomitant procedures and medications during 2nd-line therapy (yes vs no)			
Qualitative proteinuria tests	0.85	0.66–1.09	0.1879
Quantitative proteinuria tests	0.85	0.63–1.15	0.3038
Antihypertensives	0.83	0.68–1.02	0.0823
Anticholinergics	1.11	0.87–1.42	0.404
Anticoagulants	1.29	0.82–2.05	0.2732

FOLFIRI, leucovorin, fluorouracil, and irinotecan; *RAS*, rat sarcoma viral oncogene homolog; CRC, colorectal cancer; CI, confidence interval; BMI, body mass index; ADL, activities of daily living; EGFR, endothelial growth factor receptor.

715 patients who started FOLFIRI plus antiangiogenic drug as second-line and had ADL and BMI data available from baseline period before second-line and with presumed *RAS*-wild type CRC were included in this analysis.
