# Supplementary material for: Treatment sequences of patients with advanced colorectal cancer and use of second-line FOLFIRI with antiangiogenic drugs in Japan: A retrospective observational study using an administrative database
Source: PLoS One. 2021 Feb 8;16(2):e0246160. doi: 10.1371/journal.pone.0246160 (PMC7870079; doi:10.1371/journal.pone.0246160)
Supplement: S5D Table — (PDF) [file pone.0246160.s013.pdf]

S5d Table. Multivariate Cox regression analysis for the factors associated with overall treatment continuation from the start of second-line therapy to the end of all antitumor drug therapies in the FOLFIRI plus antiangiogenic drug subpopulation, for patients with presumed *RAS*-mutant CRC.

Covariate	Hazard ratio	95% CI	p-value
Designated cancer hospital (yes vs no)	1.22	1.08–1.39	0.0021
≥70 vs <70 years at start of 2nd-line therapy	1.13	1–1.27	0.046
Sex: male vs female	1.06	0.95–1.2	0.3046
Left-sided CRC (yes vs no)	0.91	0.8–1.02	0.1161
BMI ≤18.5 kg/m^2 vs >18.5 kg/m^2	1.33	1.13–1.56	0.0004
ADL (not independent vs independent)	1.27	1.04–1.55	0.0204
Oral fluoropyrimidine in previous line of therapy (yes vs no)	0.81	0.71–0.92	0.0019
Irinotecan in previous line (yes vs no)	1.31	1.06–1.62	0.0131
Duration of previous line of therapy ≥180 days vs <180 days	0.91	0.81–1.03	0.1283
Early recurrence (yes vs no)	0.7	0.58–0.86	0.0005
Concomitant procedures and medications during 2nd-line therapy (yes vs no)			
Qualitative proteinuria tests	0.69	0.61–0.79	<0.0001
Quantitative proteinuria tests	0.91	0.78–1.06	0.2378
Antihypertensives	0.91	0.81–1.02	0.1015
Anticholinergics	0.87	0.75–1.01	0.0604
Anticoagulants	0.86	0.66–1.11	0.2453

FOLFIRI, leucovorin, fluorouracil, and irinotecan; *RAS*, rat sarcoma viral oncogene homolog; CRC, colorectal cancer; CI, confidence interval; BMI, body mass index; ADL, activities of daily living; EGFR, endothelial growth factor receptor.

2,020 patients who started FOLFIRI plus antiangiogenic drug as second-line and had ADL and BMI data available from baseline period before second-line and with presumed *RAS*-mutant CRC were included in this analysis.
