# Supplementary material for: Treatment sequences of patients with advanced colorectal cancer and use of second-line FOLFIRI with antiangiogenic drugs in Japan: A retrospective observational study using an administrative database
Source: PLoS One. 2021 Feb 8;16(2):e0246160. doi: 10.1371/journal.pone.0246160 (PMC7870079; doi:10.1371/journal.pone.0246160)
Supplement: S6A Table — (PDF) [file pone.0246160.s014.pdf]

S6a Table. Multivariate Cox regression analysis for the factors associated with overall treatment continuation from the start of second-line therapy to the end of all antitumor drug therapies in the FOLFIRI plus bevacizumab population.

Covariate	Hazard ratio	95% CI	p-value
Designated cancer hospital (yes vs no)	1.15	0.99–1.34	0.0706
≥70 vs <70 years at start of 2nd-line therapy	1.08	0.93–1.24	0.307
Sex: male vs female	1.05	0.92–1.22	0.4598
Left-sided CRC (yes vs no)	0.85	0.73–0.99	0.0377
Presumed *RAS*-wild type (yes vs no)	0.71	0.6–0.83	<0.0001
BMI ≤18.5 kg/m^2 vs >18.5 kg/m^2	1.25	1.03–1.51	0.0217
ADL (not independent vs independent)	1.36	1.09–1.71	0.0066
Oral fluoropyrimidine in previous line of therapy (yes vs no)	0.8	0.67–0.96	0.0161
Irinotecan in previous line (yes vs no)	0.75	0.52–1.1	0.1411
Duration of previous line of therapy ≥180 days vs <180 days	0.92	0.8–1.06	0.2502
Early recurrence (yes vs no)	0.73	0.59–0.91	0.0053
Concomitant procedures and medications during 2nd-line therapy (yes vs no)			
Qualitative proteinuria tests	0.77	0.66–0.89	0.0006
Quantitative proteinuria tests	0.9	0.72–1.13	0.3677
Antihypertensives	0.92	0.8–1.06	0.2532
Anticholinergics	0.84	0.71–1	0.0506
Anticoagulants	0.89	0.64–1.24	0.495

FOLFIRI, leucovorin, fluorouracil, and irinotecan; CRC, colorectal cancer; CI, confidence interval; *RAS*, rat sarcoma viral oncogene homolog; BMI, body mass index; ADL, activities of daily living; EGFR, endothelial growth factor receptor.

1,476 patients who started FOLFIRI plus bevacizumab as second-line and had ADL and BMI data available from baseline period before second-line were included in this analysis.
