# Supplementary material for: Treatment sequences of patients with advanced colorectal cancer and use of second-line FOLFIRI with antiangiogenic drugs in Japan: A retrospective observational study using an administrative database
Source: PLoS One. 2021 Feb 8;16(2):e0246160. doi: 10.1371/journal.pone.0246160 (PMC7870079; doi:10.1371/journal.pone.0246160)
Supplement: S6B Table — (PDF) [file pone.0246160.s015.pdf]

S6b Table. Multivariate Cox regression analysis for the factors associated with overall treatment continuation from the start of second-line therapy to the end of all antitumor drug therapies in the FOLFIRI plus ramucirumab population.

Covariate	Hazard ratio	95% CI	p-value
Designated cancer hospital (yes vs no)	1	0.83–1.21	0.9852
≥70 vs <70 years at start of 2nd-line therapy	0.98	0.82–1.16	0.7822
Sex: male vs female	1.03	0.86–1.22	0.7704
Left-sided CRC (yes vs no)	0.83	0.69–1	0.0467
Presumed *RAS*-wild type (yes vs no)	0.66	0.53–0.82	0.0003
BMI ≤18.5 kg/m^2 vs >18.5 kg/m^2	1.47	1.16–1.87	0.0017
ADL (not independent vs independent)	1.71	1.24–2.35	0.0011
Oral fluoropyrimidine in previous line of therapy (yes vs no)	0.79	0.65–0.96	0.016
Irinotecan in previous line (yes vs no)	1.19	0.93–1.53	0.1626
Duration of previous line of therapy ≥180 days vs <180 days	0.84	0.71–1	0.055
Early recurrence (yes vs no)	0.62	0.38–1.03	0.0643
Concomitant procedures and medications during 2nd-line therapy (yes vs no)			
Qualitative proteinuria tests	0.64	0.51–0.79	<0.0001
Quantitative proteinuria tests	0.89	0.72–1.1	0.2803
Antihypertensives	0.72	0.6–0.86	0.0002
Anticholinergics	1.08	0.88–1.34	0.4519
Anticoagulants	1.08	0.76–1.54	0.6634

FOLFIRI, leucovorin, fluorouracil, and irinotecan; CRC, colorectal cancer; CI, confidence interval; *RAS*, rat sarcoma viral oncogene homolog; BMI, body mass index; ADL, activities of daily living; EGFR, endothelial growth factor receptor.

942 patients who started FOLFIRI plus ramucirumab as second-line and had ADL and BMI data available from baseline period before second-line were included in this analysis.
