# Supplementary material for: Treatment sequences of patients with advanced colorectal cancer and use of second-line FOLFIRI with antiangiogenic drugs in Japan: A retrospective observational study using an administrative database
Source: PLoS One. 2021 Feb 8;16(2):e0246160. doi: 10.1371/journal.pone.0246160 (PMC7870079; doi:10.1371/journal.pone.0246160)
Supplement: S6C Table — (PDF) [file pone.0246160.s016.pdf]

S6c Table. Multivariate Cox regression analysis for the factors associated with overall treatment continuation from the start of second-line therapy to the end of all antitumor drug therapies in the FOLFIRI plus aflibercept beta population.

Covariate	Hazard ratio	95% CI	p-value
Designated cancer hospital (yes vs no)	1.46	0.96–2.22	0.0755
≥70 vs <70 years at start of 2nd-line therapy	1.21	0.86–1.7	0.2718
Sex: male vs female	1.18	0.85–1.66	0.3222
Left-sided CRC (yes vs no)	0.96	0.69–1.35	0.8339
Presumed *RAS*-wild type (yes vs no)	0.52	0.35–0.77	0.0012
BMI ≤18.5 kg/m^2 vs >18.5 kg/m^2	1.58	1.06–2.36	0.025
ADL (not independent vs independent)	1.26	0.69–2.29	0.4484
Oral fluoropyrimidine in previous line of therapy (yes vs no)	0.63	0.44–0.9	0.0114
Irinotecan in previous line (yes vs no)	1.07	0.72–1.58	0.7374
Duration of previous line of therapy ≥180 days vs <180 days	0.87	0.63–1.2	0.384
Early recurrence (yes vs no)	0.87	0.4–1.89	0.726
Concomitant procedures and medications during 2nd-line therapy (yes vs no)			
Qualitative proteinuria tests	0.49	0.32–0.74	0.0008
Quantitative proteinuria tests	0.73	0.5–1.05	0.089
Antihypertensives	0.8	0.58–1.11	0.1856
Anticholinergics	0.78	0.53–1.13	0.1897
Anticoagulants	0.87	0.46–1.66	0.6765

FOLFIRI, leucovorin, fluorouracil, and irinotecan; CRC, colorectal cancer; CI, confidence interval; *RAS*, rat sarcoma viral oncogene homolog; BMI, body mass index; ADL, activities of daily living; EGFR, endothelial growth factor receptor.

317 patients who started FOLFIRI plus aflibercept beta as second-line and had ADL and BMI data available from baseline period before second-line were included in this analysis.
