# Supplementary material for: Treatment sequences of patients with advanced colorectal cancer and use of second-line FOLFIRI with antiangiogenic drugs in Japan: A retrospective observational study using an administrative database
Source: PLoS One. 2021 Feb 8;16(2):e0246160. doi: 10.1371/journal.pone.0246160 (PMC7870079; doi:10.1371/journal.pone.0246160)
Supplement: Attachment — Submitted filename: ResponseToReviewers.docx [file pone.0246160.s017.docx]

**Response to Comments from Peer Review**

**Editor**

*Comment 1:* As the revisers’ comments, this study is now ready to acceptance. However, there still some concerns.

*Authors’ Response*:

Thank you for your valuable feedback. We have explained below the changes made to the manuscript to address your concerns.

*Comment 2:* P20, Abstract, the sentence is difficult to follow, “Patients who underwent adjuvant therapy (and presumably experienced early recurrence) or first-line treatment for CRC in or after May 2016 were analysed until September 2019.” This mean ‘this study enrolled CRC patients who underwent first-line chemotherapy, including the patients who received adjuvant therapy but presumably experienced early recurrence from May 2016 to September 2019’?

*Authors’ Response:* The patient enrolment period was between May 2016 and July 2019, and the enrolled patients were followed up until September 2019. To clarify the sentence, we have modified the sentence to the following:

*“Patients were enrolled if they started adjuvant therapy (and presumably experienced early recurrence) or first-line treatment for advanced CRC between May 2016 and July 2019, and were analysed until September 2019.”*

*Comment 3:* P27, Treatment sequences, the following sentences are very difficult to follow. “First-line or adjuvant therapy ended when patients added a new antitumor drug not included in the treatment regimen or when patients discontinued all antitumor drugs in the treatment regimen for 180 days or more, whichever occurred first.”

*Authors’ Response:* As per your comment, we have amended the sentence to help clarify, as follows:

*“The end of first-line or adjuvant therapy was defined either as 1) discontinuation of all antitumor drugs for 180 days or more, or 2) prescription of new antitumor drugs that were not included in the first-line or adjuvant treatment regimen, whichever occurred first.”*

*Comment 4:* Table 3, Number of antiangiogenic drug prescriptions Median (IQR) 5 (3–9). Please exclude mean (SD) 6.9 (6.1).

*Authors’ Response:* Thank you for your suggestion. In Table 3, we have removed the mean (SD), and now show the median (IQR) only. Please note that we have made the same modification to S3a-c Tables.
